# Social Aspects of Tuberculosis*Address delivered before the West Texas Medical Association, October, 23, 1902.

**Published:** 1902-12

**Authors:** F. E. Daniel

**Affiliations:** Austin, Texas


### TEXAS MEDICAL JOURNAL.

ESTABLISHED JULY, 1885.

PUBLISHED MONTHLY.—SUBSCRIPTION $1.00 A YEAR.

VOL. XVIII. AUSTIN, DECEMBER, 1902. No. 6.

### Original Contributions.

### Social Aspects of Tuberculosis.*

BY F. E. DANIEL, M. D., AUSTIN, TEXAS.

*Mr. President and Gentlemen:*

On this, the twenty-sixth annual reunion of the famous West Texas Medical Association—a society whose meetings are always characterized by interest, enthusiasm and good fellowship—I, an honorary, not an active, member, feel complimented that you should have assigned me a place on your program. I confess, however, that I was surprised, upon receiving the printed program yesterday, to see that I was down for an address—and in capital letters. Our friend, Dr. Russ, your able secretary, is a "capital" fellow, but if he has led you to expect an address, "capital" or otherwise, I fear you will be disappointed. I have made no preparation whatever, and I cannot give you an address. I would have been glad of an opportunity to prepare something worthy of your attention and of the occasion, but I shall have to endeavor to hold your attention a few moments by such thoughts and facts as come to me at the moment. It is late. You are tired. You have had a good dinner and a good cigar, and I know how you feel. You have listened with patience and your usual courtesy to a number of papers and the discussion of a number of "interesting cases," and we are to have an evening session—and "after that the deluge"—no, I mean the banquet.

*Address delivered before the West Texas Medical Association, October, 23, 1902.

Hence it would be inconsiderate in me, if not unkind, to detain you.

You have given me for a subject, "The Social Aspect of Tuberculosis." In the whole range and scope of social science, as broad and comprehensive as it is, embracing every interest of the social body of the nation—its commercial, financial, industrial, scientific, religious, economical and political—there is no more important subdivision than that of the public health. It is the one vital subject, the foundation upon which rest the prosperity, the integrity, the welfare, the perpetuity—the very existence of a State or nation. It is of vastly more importance than are those conditions which affect the morals of the people—crime and prostitution, for instance, as vast and far-reaching as are their pernicious influences; or of industrial disturbances, as "strikes," etc.; or of pauperism, or of any of the problems with which the student of sociology is confronted.

In a consideration of the subject we should take into account all those things which affect the health and threaten the life of the citizen. We will limit ourselves, however, to a brief consideration of consumption in relation to the public, "The Social Aspect of Tuberculosis." It is a phase of the science that strikes at the underlying principle of the social fabric—the public health. It is the blight upon the nation—the one chief factor in death and decay—the overshadowing evil that perpetually hangs like a pall upon every people. It destroys more lives than "plague, pestilence and famine, battle, murder and sudden death." It is responsible for 16.16 per cent. of all the deaths from all causes, including war and famine. That is, about one-sixth of those who die, die of consumption, every year, everywhere, all the time! And yet, it is a preventable disease, easily preventable! What a commentary upon our civilization and boasted enlightenment! We hasten slowly. We make slow progress in preventive medicine—a progress out of all proportion to the advances made in every other line of human thought and activity.

How far-reaching and disastrous are the ravages of tuberculosis may be seen at a glance at a few figures of the statistician. Dr. W. S. Carter, of the Medical Department of the University of Texas, at Galveston, in a paper presented to the Texas State Medical Association at Dallas last April, says that in the United States there are one hundred and fifty thousand deaths from consumption every year. That is about 50 per cent. more than the total deaths in the United States from yellow fever in a century! And yet, in face of these appalling facts, the health machinery of many of the States and of the United States is directed almost solely towards the prevention of epidemic diseases of foreign origin; and nearly all of the vast amount of money spent for sanitation is expended in the endeavor to prevent the introduction of those diseases into our midst; that is, for quarantine and inspection service. One hundred and fifty thousand lives lost every year in the United States by one preventable disease! It is estimated that the money value of those lives—to say nothing of time lost, suffering, expense of treatment and nursing; that is, the value to the State of a citizen as a producer, or a soldier, if need be—is about one hundred and fifty million dollars, a sum sufficient to pay the public debt, or to build an isthmian canal every year. A hundred and fifty thousand deaths in the United States every year from consumption alone! That is about one-fourth of one per cent. of the entire population. If unchecked, at that rate, in about forty years, the population will be decimated. The world stands awed and shocked at contemplating the destruction of forty thousand people by an earthquake. It would be horrified at a war in which one hundred and fifty thousand were slain. And yet, that number of our people fall every year in these enlightened United States by tuberculosis alone, and it is scarcely known or heard of except by the sanitarian and the physician.

But at last the people are being aroused to a realizing sense of this enormous and needless loss of life; the whole medical world is awakened to the importance of devising means to suppress and eliminate this terrible scourge. Tuberculosis congresses are being held everywhere, and special sanitariums are being established for the treatment and cure of the indigent consumptive. A sentiment has been created in Texas, too, by the physicians, and our people are beginning to move to take some steps towards arresting the spread of the disease. It is hoped that at the approaching session of the Legislature the medical societies of the State will be able to secure the passage of measures looking to that end—the creation of a State Board of Health, with power to formulate and enforce measures of prevention and the establishment of consumptives' hospitals, in which the "floating population" of migratory consumptives of the pauper class that drift southward every winter, may be "corraled," "retired from circulation."

Any proposed reform, however rational, is apt to meet with opposition. Any reform that strikes at preconceived and established ideas, doctrines and customs, is necessarily of slow growth; and whenever any reform is instituted, there is not infrequently a reaction, and we are apt to go to an opposite extreme and substitute one evil for another. Hence, in the matter of dealing with the prevention of consumption, calm and deliberate judgment and good sense must prevail in order to not create alarm or panic.

In the French relovution, when the people, goaded to desperation by centuries of wrong and oppression, rose in their might, killed their king and queen, exterminated the aristocracy, and seized the government, they went to the extreme of killing each other, and inaugurated the "Reign of Terror." There is danger here of going to extremes in the endeavor to arrest the spread of consumption. In our anxiety to protect the public we may institute unnecessarily severe measures, the enforcement of which would work great hardships and be an outrage on humanity. The impression prevails very generally, unfortunately—and it is upon the authority of those who know better (but it grows out of a confusion of terms)—that consumption is contagious. It is remarkable that words are used as synonyms, that have, often, widely different meaning. "Contagious" and "infectious," "communicable" are generally accepted and used as meaning the same thing, and yet there is a difference in many ways between a "contagious" disease and a "communicable" disease. A disease may be communicable without being contagious, as are typhoid fever, cholera and consumption. But all these are communicable, and infectious in the sense that the poison thrown off by them in the secretions and excretions may and do infect, and hence communicate the disease to well persons. I repeat and wish to emphasize it: Consumption is a communicable disease, but it is in no sense contagious—that is, it cannot be "caught" by contact. That is the meaning of the word. It is not possible for a sound, healthy body, coming in contact with a consumptive body, to acquire consumption. Nevertheless, upon the authority of the Surgeon General of the Marine Hospital Service of the United States, backed by that of the United States Treasury Department, of which the Marine Hospital Service is a sub-department or bureau, the fiat has gone out that upon a construction of the public health laws by the Attorney General, consumption is "a contagious disease, dangerous to the public health." In June last the Superintendent of Immigration of the United States issued an order, based upon this decision, that all consumptive immigrants, without distinction, rich and poor, big and little, shall be excluded from the shores of the United States. Previous to this order the Board of Immigration, after having received the opinion of the health officer of the port, had some discretion, and it was possible to admit a child afflicted with consumption, accompanied by healthy parents, or a consumptive wife along with her healthy husband, for instance; but under this ruling every consumptive must be sent back. This involves the separation of parents from child, of husband from wife, the disruption of whole families. Reflect a moment upon the unwisdom of such a course, the utter needlessness of it, not to say the inhumanity and injustice of it. I have heard it seriously proposed to stop consumptives at the State line and refuse them admission to our State. I am under the impression that such a policy is in force in California. Such action would be unwise, irrational, inhuman. It would be worse than the "shot-gun quarantine" of former years. It would create a panic in the public and unjustly put a stigma upon thousands of good citizens and desirable immigrants, sufferers from an acquired disease. The consumptive is not a danger to be shunned and fled from like the plague. He is not a leper, "unclean, unclean," to be avoided upon all occasions. He can be rendered harmless by the observance of simple and rational measures, and many will recover under proper hygienic conditions and environments.

And here let me say a word about the exploded yet popular fallacy that consumption is a hereditary disease. It is not. It cannot be transmitted from parent to child, but the child may, and often does, acquire it in the way that we now know that it is communicated, by infection from the expectorated matter that contains the poison (tubercle bacilli). A predisposition to consumption may be transmitted from an invalid parent, a weakened vitality that would predispose to almost any disease, but consumption is never "inherited." The measures of prevention are: First, every case of consumption should be reported to the health officer, and should be kept under observation. The sufferer should be instructed and shown how and enjoined for his own safety and that of others, to destroy the expectorated matter that contains the poison (tubercle bacilli), and, in fact, all the excreta; to live in well-ventilated rooms; to bathe often, cold baths being preferable, they are "tonic"; to live out-of-doors as much as possible, sleep with open windows, avid drugs and stimulants, eat wholesome and nutritious food and plentifully; take frequent exercise, stopping short of extreme fatigue, etc. The rooms occupied by the consumptive should be daily opened to the sun and air, though they alone will not disinfect the room; the bacilli are very tenacious of life and can only be destroyed by chemical means or by fire. All excreta should be burned, and the cuspidors should always contain a solution of bichloride of mercury or carbolic acid. Better, small paper receptacles made for the purpose should be carried by the patient and destroyed at convenient opportunities. All sleeping apartments and sleeping cars occupied by a consumptive should be disinfected at intervals, sterilized by the well-known germicidal gases, sulphur dioxide and formaldehyde. Ever since the discovery of the tubercle bacillus by Koch we know that this germ is the source of the disease. It gets into the dust after the expectorated matter has dried, and a well person acquires the disease by inhaling the dust into his lungs, and a patient, recovering from the disease, reinfects himself. Hence, if the expectorated matter be promptly destroyed the disease will be disarmed of its dangers, and that should be done in every case. Ordinances should be enforced everywhere prohibiting spitting in public places by all persons.

These are precious truths and should be made known to the public. The people should be made to realize the danger and taught how to avoid it, and to know the deadly danger of disregarding the warnings. How can this be done? Physicians and sanitarians meet and discuss these things. They are published in the medical journals, but the public do not get the benefit of it. The lay press is very reluctant to publish such matters because the editors do not understand and appreciate the enormous importance of disseminating the information. We are like a convention of teachers holding sessions with closed doors. The pupils of the schools do not get the benefit of the teachings. One important feature of the proposed State Board of Health will be the preparation of literature on this subject and its general distribution among the people.

There is a great deal of missionary work to be done, too, in our own ranks. A great many of the rank and file of the medical profession do not understand the true nature of tuberculosis, and, therefore, do not know how to deal either with its prevention or treatment. Their minds must be disabused, too, of the "hereditary" fallacy.

Any remarks upon the treatment of the disease would scarcely come appropriately under the head of my subject, but as the greater always comprehends the lesser, it will not be amiss to say that the best thought of the medical profession of the world now is that drug treatment is useless, and worse than useless, and it has been abandoned. The materia medica, so far as it is of use in treating consumption, except as palliative in certain cases, has been relegated to the attic of exploded dogma. It is only by prevention that the disease can be controlled and finally exterminated, and by hygienic measures that it can be cured. The means necessary to the first are so simple and easily used, and enforced by authority, if necessary, that they should be instituted at once. The hope of exterminating the disease, however, is Utopian. It involves a complete change in the manner of living and in the construction of dwellings, factories, shops, railway coaches—day and sleeping. All upholstered furniture and equipment, even carpets, should be abolished in hotels, boarding houses and sleeping cars. Dwellings and all living apartments and public conveyances should be constructed, not only for comfort and convenience, but with an eye to preserving the health—the avoidance of the cause of disease—which are now very generally known to the medical men and must be taught every citizen. The means employed to prevent the spread of the disease from the sick to the well, and the reinfection of the patient himself (auto-infection), are suggested by and should be based on the etiology of the disease, and a knowledge of the manner in which it is propagated. Destroy the poison that is thrown off from the coughing consumptive, destroy the germs that linger in the dust in the furnishings and crevices of the apartments and conveyances that are, or have been, occupied by him. Consumption is not a quarantinable disease, and should not be quarantined. In public institutions, however, hospitals, penitentiaries, asylums, etc., it is best to separate the consumptive from the other inmates. The treatment of those already afflicted with the disease must be hygienic. "Sanitation is the medicine of the future," said Professor Chaille; drainage, correct plumbing, ventilation, suitable climate, pure water, fresh air—and plenty of it—sunshine, cleanliness, nutritious diet, cheerful environment, diversion, exercise, etc., and sterilized apartments; everything, in fact, that makes for health of body and mind.

It is remarkable how near we have come, in the past, to hitting on the truth and solving certain problems; this very one of consumption amongst them. When King Edward VII ordered the founding of the King's Sanitarium for Indigent Consumptives, a prize of $2,500 was offered for the best essay upon consumption and the management and treatment of the disease, and its prevention. Dr. Arthur Latham was the successful competitor. He advocates the hygienic method of treatment and taboos drugs. Says a writer in *The Hospital* (a medical magazine published in London), speaking of the subject:

"It is one of our amiable weaknesses to hold patent medicines in ridicule and contempt, but what could be more ridiculous, considering the teachings of the dead-house, than the current treatment of consumption so aptly described by Dr. Latham—a mere pouring in of drugs without any attempt to touch the root of the disease. Yet in the midst of all this drugging, which has been going on far longer than we can remember, there have been men who saw the truth.

"So far back as 1840, George Bodington insisted on the importance of a generous diet and a constant supply of pure air, and propounded the terrible heresy that 'cold is never too intense for a consumptive patient.' In 1855 Dr. Henry MacCormac, the father of the late Sir William MacCormac, published a book on somewhat similar lines, and in 1861 read a paper before the Royal Medical and Chirurgical Society in which he advocated what are now established principles. Yet what was the treatment which these pioneers received at the hands of their professional colleagues? 'Bodington's book,' says Latham, 'met with much bitter and fierce opposition, and eventually the disapproval of his methods became so universal that patients were driven from his sanitarium,' while 'the members of the Royal Medical and Chirurgical Society refused to pass the usual vote of thanks to Dr. MacCormac, because they thought that the paper was written by a monomaniac.' * * * Meanwhile, notwithstanding our ostracism of new ideas, the teaching of Bodington, of MacCormac, and of the modern host of sanitarium owners has prevailed; and now, at last, in the full sunshine of royal patronage, we admit how simple is the truth expressed as it is by the motto of Dr. Latham's essay: 'Give him air; he'll straight be well.' What sycophants we all are!"

That sets the pace. The King's Sanitarium sets the precedent. The world will follow. The treatment of consumption for the future must be hygienic, sanitation and ventilation, and our people are slowly awakening to this; we must arouse them. The more enlightened amongst the people are beginning to understand that fresh air, and the "bugaboo" "night-air" is not injurious, and that cold water is not poisonous.

